# Global research landscape and emerging frontiers of ultra-processed food intake and cardiometabolic health: a bibliometric and knowledge-mapping analysis (2015–2025)

**DOI:** 10.3389/fnut.2026.1916253

**Published:** 2026-10-07

**Authors:** Tianao Chen, Ping Ni, Minxin Li, Haijun Ma, Jin Dai, You Yu, Rui Yu

**Affiliations:** 1 Liaoning University of Traditional Chinese Medicine, Shenyang, Liaoning, China; 2 Liaoning University of Traditional Chinese Medicine Affiliated Second Hospital, Shenyang, Liaoning, China

**Keywords:** bibliometric, cardiometabolic diseases, nutrition, public health, ultra-processed foods

## Abstract

**Objective:**

Bibliometric and visual analysis of research on ultra-processed food (UPF) intake and cardiometabolic health from 2015 to 2025.

**Methods:**

Publications from 2015 to 2025 were retrieved from the Web of Science Core Collection (WoSCC) and Scopus databases. Analyses were conducted using R software (bibliometrix package), VOSviewer, CiteSpace, and Origin 2018.

**Results:**

Scholarly output in this field demonstrated a continuous growth trajectory throughout the study period, with the steepest rise concentrated between 2020 and 2021. Brazil led in publication output, followed by the United States and China. The University of São Paulo ranked first among contributing institutions. *Nutrients*, *Public Health Nutrition*, and *Frontiers in Nutrition* were the most productive journals, while *Public Health Nutrition*, *Nutrients*, and the *American Journal of Clinical Nutrition* dominated the co-citation network. Prominent keywords included obesity, cardiovascular diseases, children, dietary patterns, and diabetes mellitus, reflecting the central themes of this field. Bibliometric analysis identified three key research hotspots: the association between UPF intake and cardiometabolic diseases (CMDs); the association between UPF consumption and early cardiometabolic impairment in children and adolescents; and mechanisms underlying food matrix disruption and food additive-mediated cardiometabolic dysregulation.

**Conclusion:**

Global research interest in the relationship between UPF intake and cardiometabolic health has grown substantially over the past decade. This study provides a comprehensive mapping of the current research landscape, key hotspots, and emerging frontiers in this field, offering a valuable reference for future investigations and public health interventions.

## 1 Introduction

Ultra-processed foods (UPFs) were first conceptualized by Monteiro and colleagues at the University of São Paulo, Brazil, in 2009. The NOVA classification system categorizes all foods into four groups: unprocessed or minimally processed foods, processed culinary ingredients, processed foods, and UPFs (1, 2). NOVA has been widely applied in nutritional epidemiology and public health research to investigate the associations of UPF intake with diet quality and its potential effects on human health (3, 4). UPFs are industrially formulated products derived primarily from food-source extracts and industrially synthesized substances, typically containing emulsifiers, artificial sweeteners, flavorings, colorants, and other food additives, and are specifically designed to maximize palatability, convenience, and consumer appeal (5). Compared with unprocessed foods, UPFs generally exhibit higher energy density and higher levels of saturated fat, added sugars, and sodium, while providing substantially lower amounts of dietary fiber, vitamins, and other protective nutrients (6). In recent decades, UPF consumption has risen markedly worldwide. In high-income countries such as the United States, Canada, the United Kingdom, and Australia, UPFs now account for a substantial proportion of total dietary energy intake (7, 8). Concurrently, sales of UPFs are growing rapidly across many middle-income countries (9). Given their widespread consumption and potential adverse health effects, UPFs have emerged as a major focus of contemporary nutritional science and public health research.

Against the backdrop of rising UPF consumption, the association between UPFs and cardiometabolic diseases (CMDs) has attracted increasing scientific attention. CMDs encompass a cluster of conditions defined by cardiovascular, metabolic, and inflammatory dysregulation, including obesity, type 2 diabetes mellitus (T2DM), hypertension, dyslipidemia, metabolic syndrome, and atherosclerotic cardiovascular disease (CVD) (10–13). These conditions share substantial pathophysiological overlap, commonly manifesting as chronic low-grade inflammation, impaired glucose and lipid metabolism, oxidative stress, and endothelial dysfunction, and collectively represent a growing global public health burden (14–18). Extensive epidemiological and interventional evidence indicates that high UPF intake is closely associated with elevated risks of obesity, insulin resistance, T2DM, metabolic syndrome, and cardiovascular events (19–24). The underlying mechanisms likely involve multiple pathways, including gut microbiota dysbiosis, toxic effects of food additives, overconsumption driven by ultra-high energy density, and nutrient displacement effects.

Although the volume of research on UPFs and cardiometabolic health has expanded rapidly in recent years, existing reviews have predominantly focused on specific mechanistic pathways or individual cardiometabolic disease outcomes, with limited integrative analysis. However, studies using bibliometric analysis and visualized knowledge mapping to systematically characterize the overall knowledge structure, evolution of research hotspots, and emerging frontiers of this field from 2015 to 2025 remain scarce. Given that the UPF concept was established within the NOVA system and that NOVA is widely used in epidemiological research to identify and classify UPFs (3, 4, 6, 7), the present study adopted NOVA as the principal conceptual framework for interpreting the retrieved literature and mapping the knowledge structure of this field. The present study therefore aims to characterize the developmental trajectory, research hotspots, and frontier directions of UPF–CMD research through a comprehensive bibliometric analysis, complemented by an integrative synthesis of mechanistic evidence, to elucidate the roles of UPFs in the onset and progression of CMDs and identify potential intervention strategies. These findings are intended to provide a theoretical foundation and research directions for future basic research, clinical translation, and public health interventions.

## 2 Data sources and methods

Bibliometric analysis is a methodological approach that applies mathematical and statistical techniques to comprehensively evaluate publications on a defined topic over a specific time period, integrating both quantitative and qualitative perspectives (25). This data-driven framework characterizes the current status, research hotspots, developmental trajectories, and emerging frontiers of a given field by examining publication output trends, contributions from countries and institutions, author collaboration networks, journal influence, citation and co-citation structures, and keyword co-occurrence and burst patterns (26). With recent advances in scientometrics, visualization techniques, and big-data processing technologies, bibliometric analysis has been widely adopted across medicine and the life sciences to map knowledge landscapes, identify research gaps, and inform strategic decision-making.

### 2.1 Data sources

Data for this study were retrieved from the Web of Science Core Collection (WoSCC) and the Scopus database, covering the period from 2015 to 2025. The full search strategies are provided in Supplementary Appendix 1. All searches were conducted on March 19, 2026. For both database searches, document types were restricted to articles and review articles, and the language filter was set to English. Following the PRISMA guidelines, two independent researchers screened the retrieved records to exclude irrelevant studies, and disagreements were resolved through discussion with the involvement of a third researcher. The remaining records were saved in plain text format and exported as complete records with full reference lists. Duplicate records were identified and removed based on DOI and title. The screening process is illustrated in the flow diagram in Figure 1. Considering that merging records from different databases may lead to inconsistencies or potential loss of critical information, including cited references and citation counts, which could affect subsequent bibliometric analyses, the WoSCC and Scopus datasets were analyzed separately to preserve data integrity. The primary bibliometric analyses were performed based on the WoSCC dataset. The Scopus dataset was used for complementary analyses, including annual publication trends, corresponding-author country distribution, and keyword co-occurrence clustering. The results obtained from both databases were subsequently comprehensively interpreted to identify research hotspots and emerging trends in this field.

**Figure 1:**
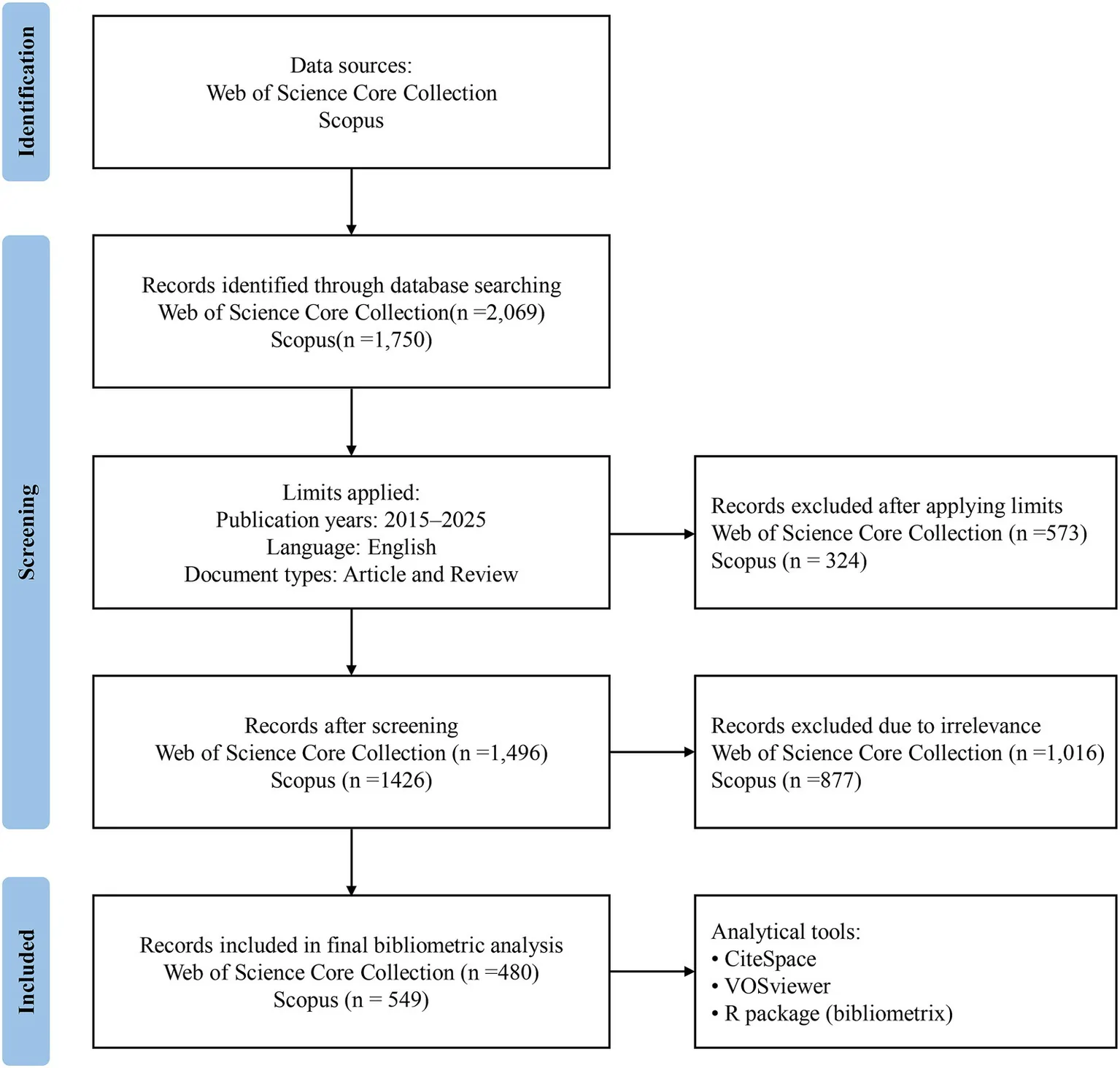
PRISMA flow diagram.

### 2.2 Data analysis methods

Temporal trends in annual publication output were examined using Origin 2018. Bibliometric assessment and knowledge visualization were carried out using the bibliometrix package (v5.0.1) in R (v4.5.1), VOSviewer (v1.6.20), and CiteSpace (v6.4). Specifically, bibliometrix was used to quantify the publication contributions of different countries, institutions, and journals; identify the most-cited references; calculate keyword frequencies; and conduct trend-topic analysis. The resulting figures were generated using the ggplot2 package in R. VOSviewer (v1.6.20) was specifically used to construct and visualize country and institutional collaboration networks, journal co-citation networks, and keyword co-occurrence networks. CiteSpace (v6.4) was used to identify references with strong citation bursts and analyze the duration of these bursts. Journal impact factor (IF) data were sourced from the 2026 Journal Citation Reports (JCR) released by Clarivate on June 17, 2026.

For the VOSviewer analyses, the following thresholds were applied to the WoSCC dataset: a minimum of 5 publications per country for the country collaboration network, a minimum of 4 publications per institution for the institutional collaboration network, and a minimum of 20 citations per journal for the journal co-citation network. For keyword co-occurrence analysis, minimum occurrence thresholds of 4 and 10 were set for the WoSCC and Scopus datasets, respectively.

To prevent keyword clusters from being dominated by search terms, major query keywords (e.g., “ultra-processed food”) and non-specific high-frequency terms (e.g., “humans,” “health,” “disease”) were excluded from the keyword co-occurrence analysis. In addition, keywords with identical or similar meanings, including synonyms, referring to the same concept, and duplicate keywords with different singular and plural forms, were standardized and merged before clustering analysis. This preprocessing step was performed to improve keyword consistency, reduce redundancy, and enhance the accuracy and representativeness of the resulting keyword clusters.

## 3 Results

### 3.1 Overview of included publications

After applying the full screening procedure, 480 eligible documents were ultimately retained from the WoSCC. As illustrated in Figure 2A, scholarly output in this field followed a consistent upward trajectory over the study period (2015–2025), with the sharpest acceleration observed between 2020 and 2021. Publications reached their decadal peak in 2025, amounting to 108 articles. In parallel, 549 unique documents were retrieved from the Scopus database. The overall growth trajectory in Scopus was highly consistent with that observed in WoSCC: in addition to the marked increase between 2020 and 2021, Scopus also showed a notable rise in publications between 2022 and 2023 (Supplementary Figure S1). This sustained upward trend reflects growing scholarly interest in this field and underscores the increasing research attention directed toward the relationship between UPF intake and cardiometabolic health.

**Figure 2:**
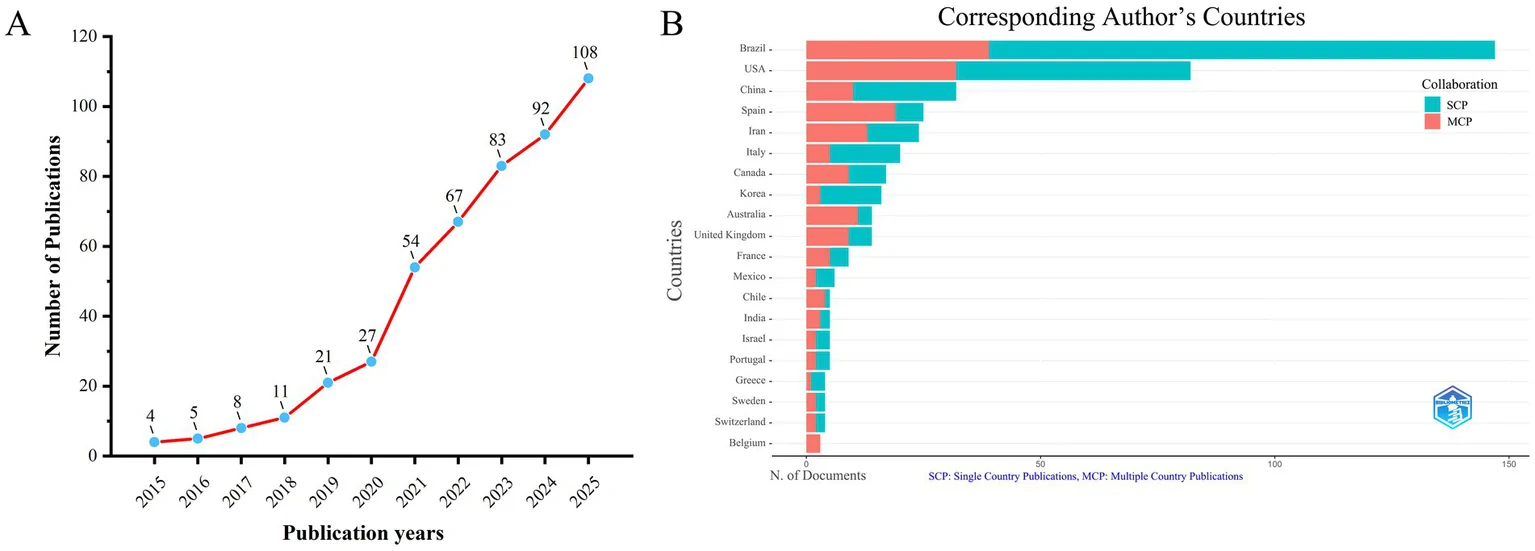
Trends in annual publications in the field of ultra-processed food intake and cardiometabolic health, 2015–2025. **(A)** Trends in publishing outputs, by year. **(B)** Distribution of corresponding authors’ countries and cooperation.

Analysis of corresponding author countries revealed that Brazil led in publication output, contributing 147 articles, followed by the United States (*n* = 82), China (*n* = 32), Spain (*n* = 25), and Iran (*n* = 24). Regarding international collaboration, Brazil (*n* = 39) and the United States (*n* = 32) emerged as the dominant hubs of the global co-authorship network among the top ten countries, reflecting their substantial overall publication volumes. Notably, European and Oceanian countries demonstrated a strong propensity for international collaboration, with Australia recording the highest international collaboration rate at 78.6%, followed by Spain (76.0%) and the United Kingdom (64.3%) (Figure 2B; Table 1). Complementary analysis of the Scopus dataset revealed a geographical distribution broadly consistent with that observed in the WoSCC dataset (Supplementary Figure S2). Brazil remained the most productive country, contributing 144 publications, followed by the United States (*n* = 86) and China (*n* = 31).

**Table 1:** Most relevant countries by corresponding authors of the relationship.

No.	Country	Articles (*n*)	Articles %	SCP	MCP	MCP %
1	Brazil	147	30.6	108	39	26.5
2	USA	82	17.1	50	32	39.0
3	China	32	6.7	22	10	31.3
4	Spain	25	5.2	6	19	76.0
5	Iran	24	5	11	13	54.2
6	Italy	20	4.2	15	5	25.0
7	Canada	17	3.5	8	9	52.9
8	Korea	16	3.3	13	3	18.8
9	Australia	14	2.9	3	11	78.6
10	United Kingdom	14	2.9	5	9	64.3

MCP, multiple country publication; SCP, single country publication.

As illustrated in Figure 3A, Brazil and the United States maintained extensive collaborative ties with other countries in the field of UPF intake and cardiometabolic health. Figure 3B and Table 2 show that among the top 20 most productive institutions, Brazil (*n* = 7) and the United States (*n* = 7) jointly dominated, followed by Iran (*n* = 3), Spain (*n* = 2), and Australia (*n* = 1). The University of São Paulo ranked first with 89 publications, an output more than twice that of the second-ranked institution, the Harvard T. H. Chan School of Public Health.

**Figure 3:**
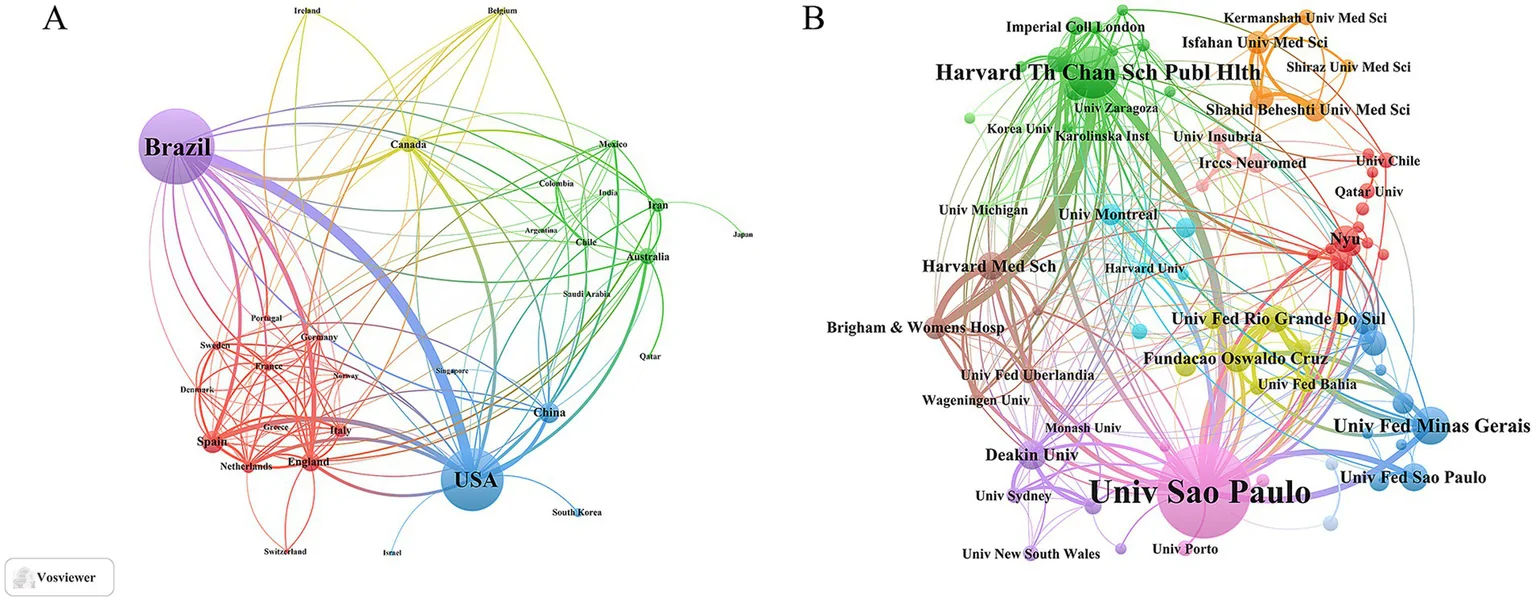
Map of cooperation between different countries/regions in the field of ultra-processed food intake and cardiometabolic health, 2015–2025. **(A)** Map of cooperation between different countries/regions. **(B)** Map of cooperation between different institutions.

**Table 2:** Top 20 most productive institutions based on corresponding-author affiliations.

No.	Affiliation	Country	Articles (*n*)
1	Universidade de São Paulo (University of São Paulo)	Brazil	89
2	Harvard T.H. Chan School of Public Health	USA	38
3	Universidade Federal de Minas Gerais (Federal University of Minas Gerais)	Brazil	23
4	Deakin University	Australia	16
5	Fundação Oswaldo Cruz (Oswaldo Cruz Foundation)	Brazil	15
6	Harvard Medical School	USA	15
7	Universidade Federal de São Paulo (Federal University of São Paulo)	Brazil	15
8	New York University	USA	14
9	Universidade Federal do Rio Grande do Sul (Federal University of Rio Grande do Sul)	Brazil	14
10	Johns Hopkins University	USA	13
11	Universidad de Navarra (University of Navarra)	Spain	13
12	Johns Hopkins Bloomberg School of Public Health	USA	12
13	Tehran University of Medical Sciences	Iran	12
14	Brigham and Women’s Hospital	USA	11
15	Instituto de Salud Carlos III (Carlos III Health Institute)	Spain	11
16	Isfahan University of Medical Sciences	Iran	11
17	Shahid Beheshti University of Medical Sciences	Iran	11
18	Tufts University	USA	10
19	Universidade Federal do Rio de Janeiro (Federal University of Rio de Janeiro)	Brazil	10
20	Universidade Federal de Viçosa (Federal University of Viçosa)	Brazil	10

### 3.2 Publication journals and co-cited journals

To identify the journals with the highest publication output and citation contribution in this field, the bibliometrix package in R was employed for quantitative assessment, and graphical outputs were rendered through the ggplot2 package. Co-citation relationships among journals were subsequently mapped using VOSviewer.

Within the WoSCC dataset, the 480 included publications were distributed across 172 academic journals. As shown in Figure 4A and Table 3, *Nutrients* (*n* = 51; IF = 5.8) published the greatest number of relevant articles, followed by *Public Health Nutrition* (*n* = 22; IF = 3.0) and *Frontiers in Nutrition* (*n* = 18; IF = 5.5). In terms of citation counts, *Public Health Nutrition* received the highest number of citations (*n* = 1,850; IF = 3.0), followed by *Nutrients* (*n* = 1,487; IF = 5.8) and the *American Journal of Clinical Nutrition* (*n* = 1,089; IF = 7.1) (Figure 4B; Table 4). As illustrated in Figure 5, the co-cited journal map identified *Public Health Nutrition, Nutrients*, and the *American Journal of Clinical Nutrition* as the structural cores of the co-citation network.

**Figure 4:**
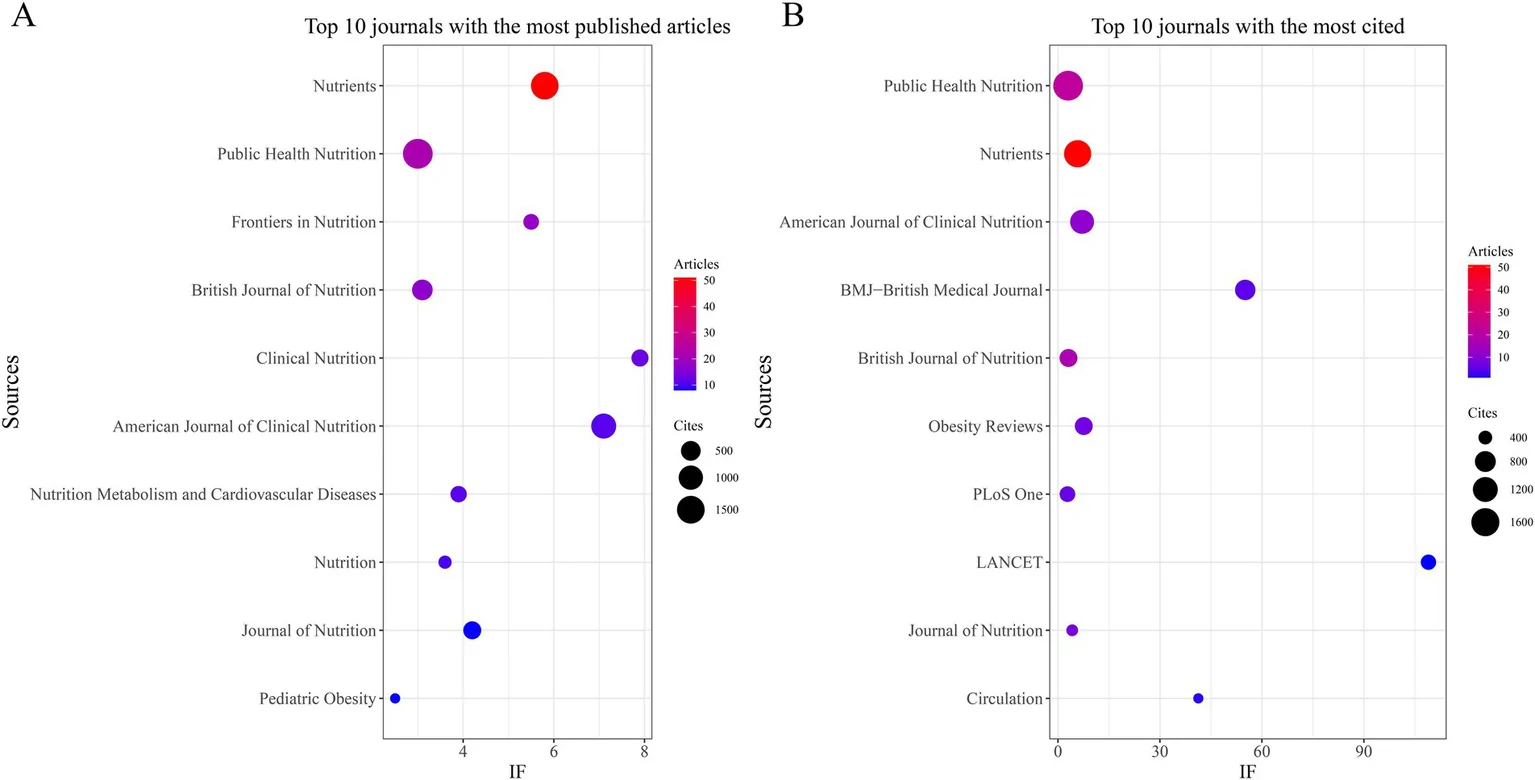
Journals with the largest number of articles published and the journal with the largest number of citations. **(A)** Journals with the most published articles. **(B)** Most-cited journals.

**Table 3:** Top 10 journals with the most published articles.

Journals	Articles (*n*)	Citations (*n*)	IF (2026)
Nutrients	51	1,487	5.8
Public Health Nutrition	22	1850	3.0
Frontiers in Nutrition	18	222	5.5
British Journal of Nutrition	17	584	3.1
Clinical Nutrition	13	300	7.9
American Journal of Clinical Nutrition	11	1,089	7.1
Nutrition Metabolism and Cardiovascular Diseases	11	253	3.9
Nutrition	10	120	3.6
Journal of Nutrition	8	360	4.2
Pediatric Obesity	8	79	2.5

**Table 4:** Top 10 most-cited journals.

Journals	Citations (*n*)	Articles (*n*)	IF (2026)
Public Health Nutrition	1850	22	3.0
Nutrients	1,487	51	5.8
American Journal of Clinical Nutrition	1,089	11	7.1
BMJ	759	5	55.1
British Journal of Nutrition	584	17	3.1
Obesity Reviews	583	7	7.6
PLoS One	468	6	2.8
LANCET	455	1	109.0
Journal of Nutrition	360	8	4.2
Circulation	351	2	41.3

**Figure 5:**
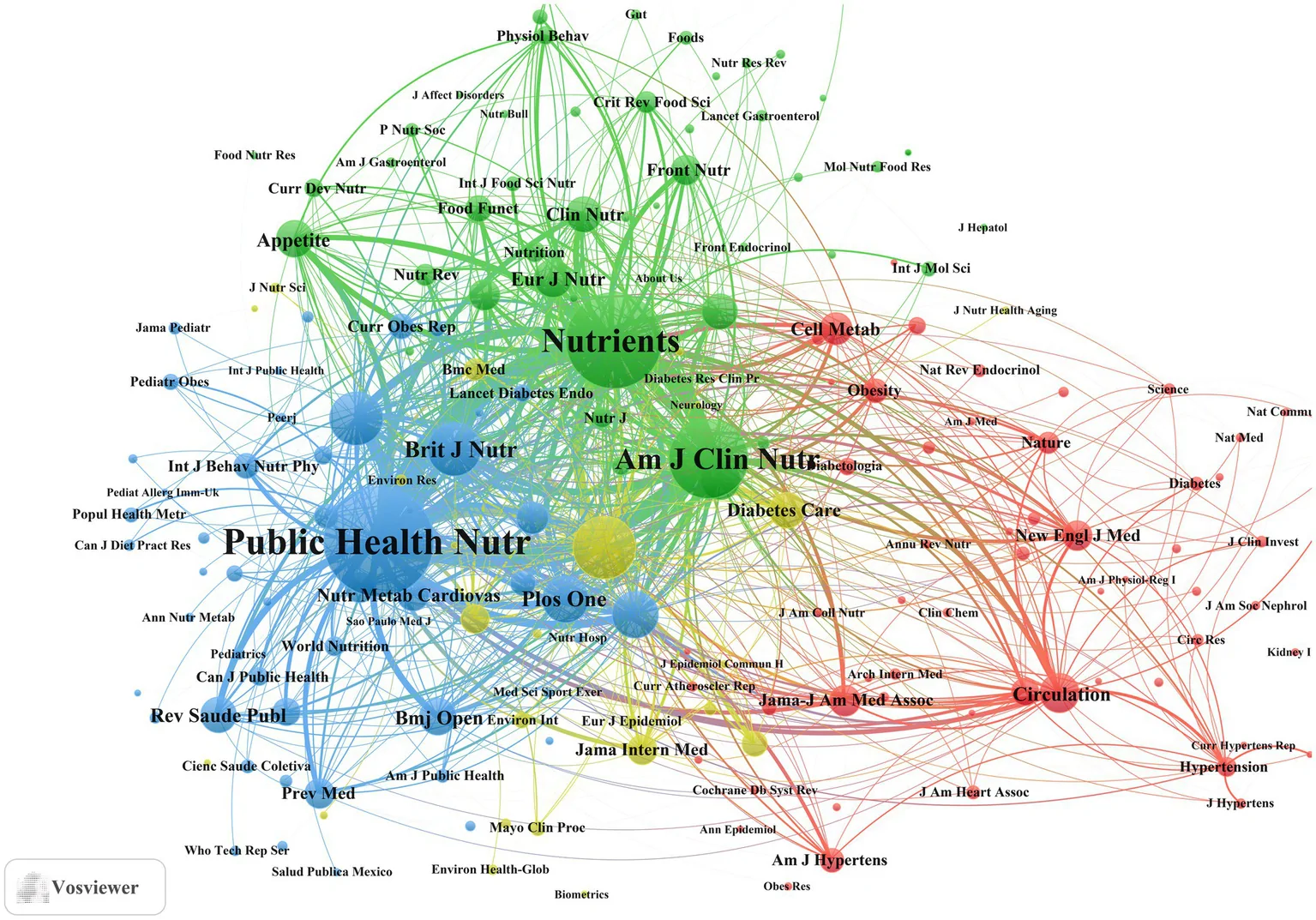
Co-cited journals involved in the field of ultra-processed food intake and cardiometabolic health.

These findings indicate that the journals listed above exert considerable academic influence in the field of UPF intake and cardiometabolic health. A noteworthy convergence was observed between journals ranking highest in publication volume and those accumulating the greatest citation impact, indicating that these outlets simultaneously function as leading platforms for emerging research and as cornerstones of the field’s accumulated knowledge.

### 3.3 Most-cited references and citation burst analysis

To explore the frontier developments and research directions in this field, the top 20 most-cited references were identified using the bibliometrix package in R. As presented in Table 5, these articles had received at least 339 citations and were published across 17 academic journals. This broad source diversity suggests that landmark research in this field is inherently multidisciplinary, necessitating continued investigation across a wider range of dimensions. Notably, the *BMJ*, *Nutrients*, and *Obesity Reviews* each contributed two articles to the top 20 most-cited list, indicating a high degree of scholarly influence for these journals within the field; however, no single journal has yet established absolute dominance among highly cited publications in this area.

**Table 5:** Top 20 most-cited references.

No.	Paper	DOI	Total citations (*n*)	TC per year
1	SWINBURN BA, 2019, LANCET	10.1016/S0140-6736(18)32822-8	2040	255.00
2	HALL KD, 2019, CELL METAB	10.1016/j.cmet.2019.05.008	1,288	161.00
3	LICHTENSTEIN AH, 2021, CIRCULATION	10.1161/CIR.0000000000001031	746	124.33
4	PAGLIAI G, 2021, BRIT J NUTR	10.1017/S0007114520002688	739	123.17
5	SROUR B, 2019, BMJ	10.1136/bmj.l1451	686	85.75
6	LANE MM, 2024, BMJ	10.1136/bmj-2023-077310	587	195.67
7	ELIZABETH L, 2020, NUTRIENTS	10.3390/nu12071955	539	77.00
8	MOUBARAC JC, 2017, APPETITE	10.1016/j.appet.2016.11.006	492	49.20
9	LANE MM, 2021, OBES REV	10.1111/obr.13146	478	79.67
10	LOUZADA MLD, 2015, PREV MED	10.1016/j.ypmed.2015.07.018	460	38.33
11	MENDONÇA RD, 2016, AM J CLIN NUTR	10.3945/ajcn.116.135004	450	40.91
12	RAUBER F, 2018, NUTRIENTS	10.3390/nu10050587	446	49.56
13	POPKIN BM, 2022, OBES REV	10.1111/obr.13366	375	75.00
14	CHEN XJ, 2020, NUTR J	10.1186/s12937-020-00604-1	374	53.43
15	MONTEIRO CA, 2018, PUBLIC HEALTH NUTR	10.1017/S1368980017001379	369	41.00
16	RAUBER F, 2015, NUTR METAB CARDIOVAS	10.1016/j.numecd.2014.08.001	356	29.67
17	MENDONÇA RD, 2017, AM J HYPERTENS	10.1093/ajh/hpw137	356	35.60
18	SROUR B, 2020, JAMA INTERN MED	10.1001/jamainternmed.2019.5942	355	50.71
19	BARALDI LG, 2018, BMJ OPEN	10.1136/bmjopen-2017-020574	340	37.78
20	ASKARI M, 2020, INT J OBESITY	10.1038/s41366-020-00650-z	339	48.43

TC, total citations.

The three most-cited articles were *“The Global Syndemic of Obesity, Undernutrition, and Climate Change: The Lancet Commission Report”* (*n* = 2,040), *“Ultra-Processed Diets Cause Excess Calorie Intake and Weight Gain: An Inpatient Randomized Controlled Trial of Ad Libitum Food Intake”* (*n* = 1,288), and *“2021 Dietary Guidance to Improve Cardiovascular Health: A Scientific Statement From the American Heart Association”* (*n* = 746). These three articles represent not merely overviews of the field, but rather exemplify three distinct research paradigms: a large-scale public health epidemiological report, a clinical randomized controlled trial (RCT), and an authoritative dietary guideline for cardiovascular health, respectively.

To further characterize prominent citation bursts in the UPF–cardiometabolic health literature, CiteSpace was employed to identify 25 references with significant citation bursts. As shown in Figure 6, the three references with the highest burst strength were *“Ultraprocessed food consumption and risk of overweight and obesity: the University of Navarra Follow-Up (SUN) cohort study”* (Strength = 26.78), *“Consumption of ultra-processed food products and its effects on children’s lipid profiles: a longitudinal study”* (Strength = 21.91), and *“Ultra-Processed Food Consumption and the Incidence of Hypertension in a Mediterranean Cohort: The Seguimiento Universidad de Navarra Project”* (Strength = 18.75). The three most recently emergent articles were *“Consumption of ultra-processed foods and cancer risk: results from NutriNet-Santé prospective cohort,” “Ultra-processed Food Intake and Obesity: What Really Matters for Health—Processing or Nutrient Content?” and “The share of ultra-processed foods determines the overall nutritional quality of diets in Brazil.”* Taken together, the analysis of highly cited references and citation burst patterns identified four recurrent thematic clusters in the UPF–CMD field: (1) UPFs influence the onset and progression of CMDs through the mediating effects of overweight and obesity; (2) the association between UPF consumption and the incidence of specific CMD endpoints; (3) UPF-induced metabolic risk through alterations in dietary nutrient composition; and (4) the early cardiometabolic harms of UPF consumption in children and adolescents.

**Figure 6:**
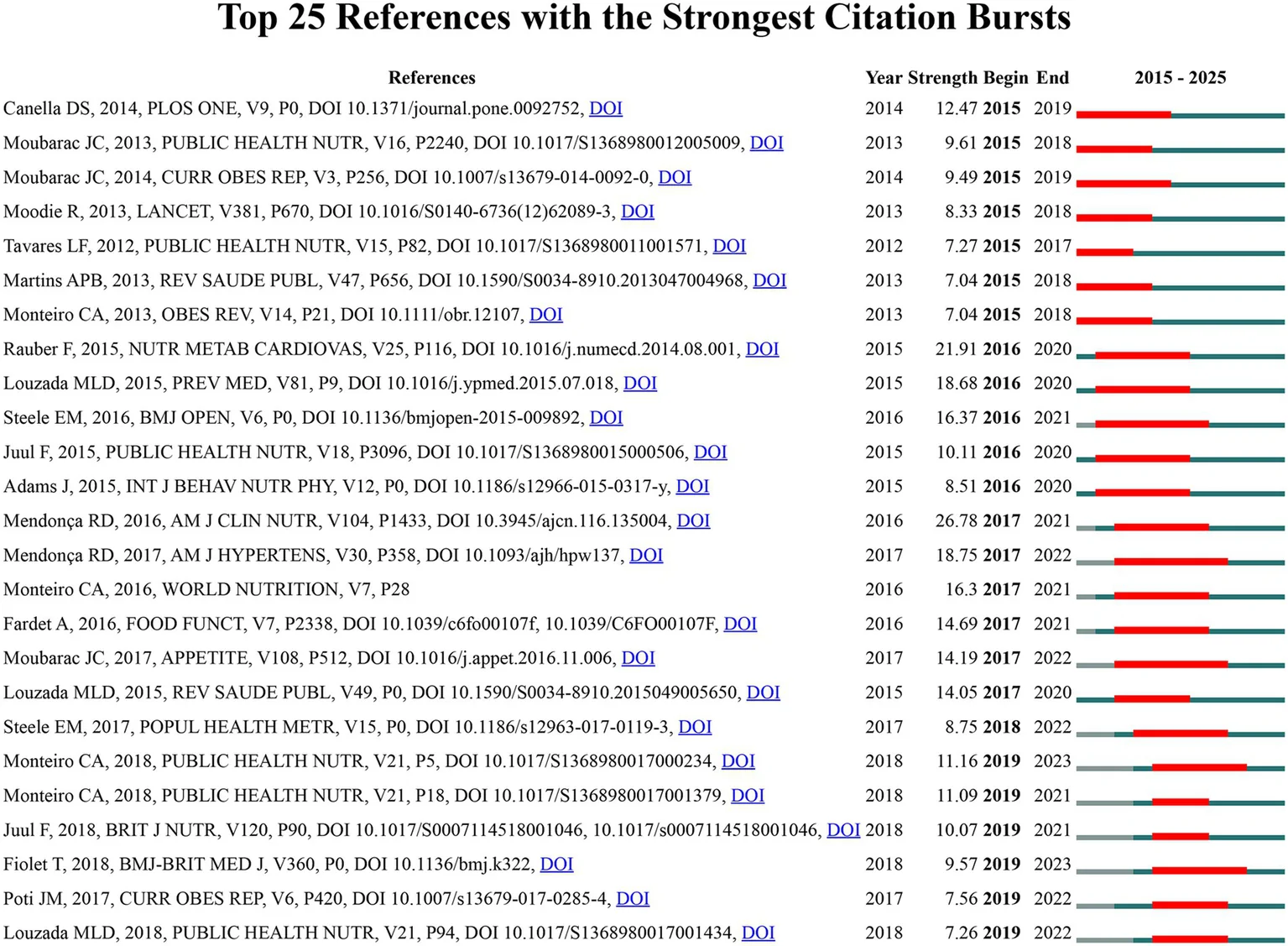
Top 25 references with the strongest citation bursts.

### 3.4 Keyword co-occurrence clustering and temporal evolution analysis

Keyword co-occurrence analysis is a widely used tool for rapidly identifying major research themes and directions within a specific academic field. A total of 1,594 keywords were extracted from the WoSCC dataset. Table 6 lists the top 20 keywords with a frequency of ≥ 30 occurrences, among which the most frequent were “obesity” (*n* = 240), “consumption” (*n* = 156), “cardiovascular diseases” (*n* = 129), “nutrition” (*n* = 86), and “children” (*n* = 78). Keywords with a minimum occurrence threshold of 4 were visualized using VOSviewer, generating a keyword co-occurrence map (Figure 7) that comprised five distinct clusters, each represented by a different color. Cluster 1 (red nodes; 40 keywords) focused on the association between UPF exposure and overweight/obesity in children and adolescents, with representative keywords including *obesity*, *children*, *adolescents*, *overweight*, and *food intake*. Cluster 2 (green nodes; 38 keywords) addressed the role of UPFs in dietary patterns and their impact on chronic disease risk, with representative keywords including nutrition, *all-cause mortality*, *dietary patterns*, *Mediterranean diet*, and *physical activity*. Cluster 3 (blue nodes; 32 keywords) encompassed the pathophysiological mechanisms underlying UPF-induced cardiometabolic dysregulation, with representative keywords including *cardiovascular diseases*, *hypertension*, *metabolic syndrome*, blood pressure, *insulin resistance*, *inflammation*, *gut microbiota*, *atherosclerosis*, and *oxidative stress*. Cluster 4 (yellow nodes; 20 keywords) covered UPF-associated glucose metabolic disturbances and diabetes risk in the context of nutritional transition, with representative keywords including *diabetes mellitus*, diet quality, sugars, *nutrition transition*, *artificial sweeteners*, *glucose,* and *glycation end-products*. Cluster 5 (purple nodes; 12 keywords) captured research on dietary assessment, classification frameworks, and nutritional intervention policies for UPFs in the context of cardiometabolic health, with representative keywords including *consumption*, *adults*, *quality*, *nutrient profile*, *dietary energy density*, and *food-processing industry*.

**Table 6:** Top 20 keywords by occurrence frequency.

Rank	Keywords	Occurrences
1	obesity	240
2	consumption	156
3	cardiovascular diseases	129
4	nutrition	86
5	children	78
6	adults	72
7	all-cause mortality	69
8	dietary patterns	67
9	adolescents	63
10	overweight	60
11	quality	55
12	diabetes mellitus	54
13	hypertension	44
14	diet quality	39
15	metabolic syndrome	39
16	food intake	37
17	body mass index	33
18	cohort studies	33
19	body weight	31
20	food processing	30

**Figure 7:**
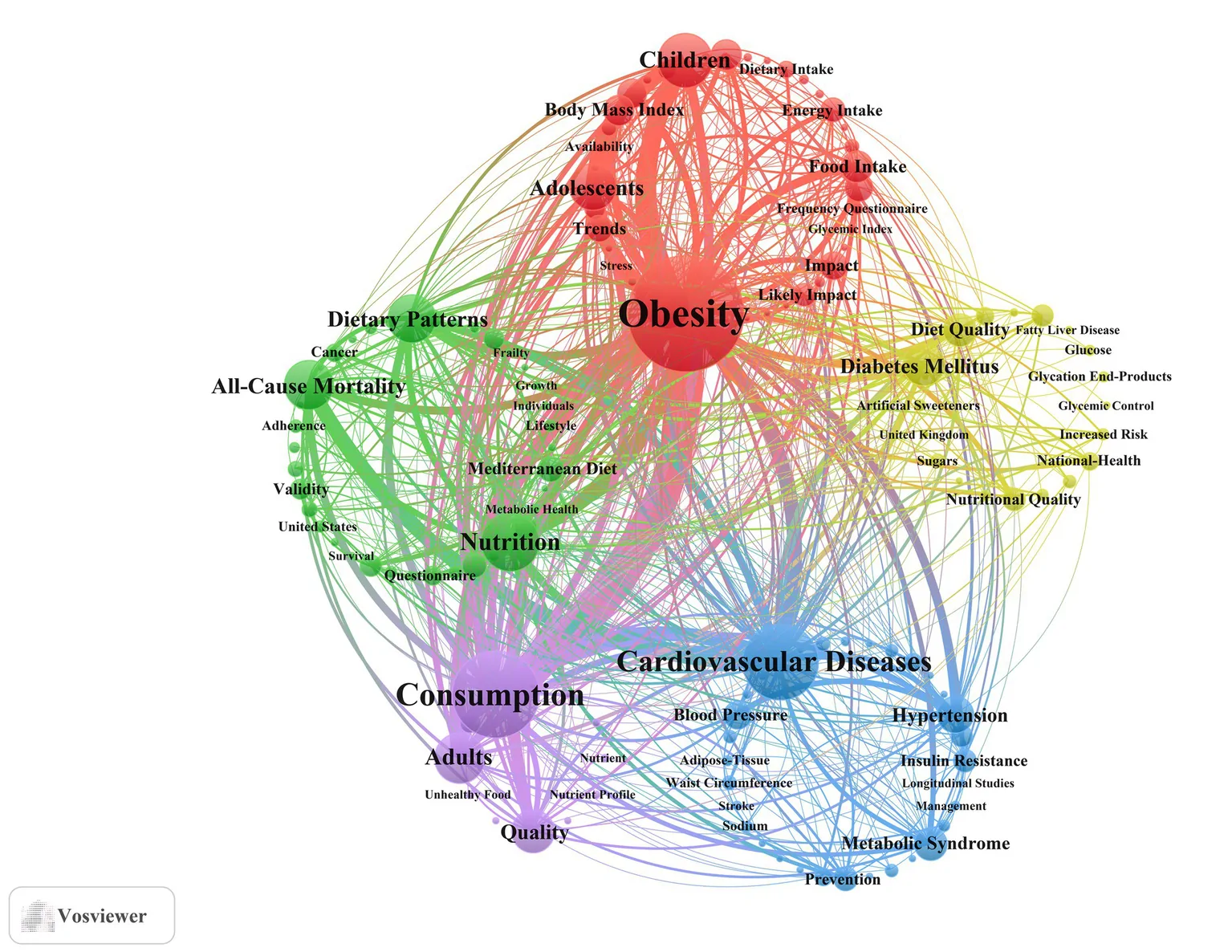
Keyword co-occurrence map of publications.

Parallel keyword co-occurrence analysis of the Scopus dataset identified four key research domains (Supplementary Figure S3): (1) the association between UPF intake and dietary patterns, nutritional composition, and diet quality; (2) UPF-related energy metabolic dysregulation and obesity-associated cardiometabolic risk; (3) the association between UPFs and the onset and progression of CMDs, including underlying pathological mechanisms; and (4) epidemiological investigations and population-level risk assessment of UPF-associated CMD risk. All keywords within these four clusters are presented in the Supplementary Table S1.

Thematic progression within this field was further explored by generating a topic evolution map through the bibliometrix package in R (Figure 8). During the period from 2016 to 2019, the field remained at an exploratory stage of development. Prominent terms included “prospective studies,” “drink products,” “likely impact,” “child nutrition,” “pregnant women,” “share,” and “risk factor,” indicating that early research primarily focused on the preliminary characterization of UPF-related dietary exposures and began to examine the potential links between their dietary share, specific product categories, and cardiometabolic risk through prospective studies. Between 2020 and 2021, the thematic emphasis gradually shifted toward the health burden, nutritional composition, and assessment methods associated with UPFs. Representative terms included “burden,” “unhealthy food,” “macronutrients,” “nutritional quality,” “national-health,” “validation,” and “products,” reflecting increasing efforts to systematically evaluate the nutritional attributes and public health implications of UPFs. From 2022 to 2023, “ultra-processed foods,” “obesity,” “risk factors,” and “cardiovascular diseases” emerged as the most frequent and representative terms, while “diets,” “food intake,” “overweight,” and “quality” remained prominent. During this period, the research focus shifted from the early characterization of dietary exposure and nutritional quality toward the systematic evaluation of associations between UPF intake and obesity, overweight, cardiovascular diseases, and related risk factors. In the most recent period from 2024 to 2025, research themes extended further toward abnormalities in glucose metabolism, disease prevention, and a broader range of health outcomes. “Dietary patterns,” “diabetes mellitus,” “prevention,” and “insulin resistance” constituted the most prominent terms during this period, indicating that the field was moving beyond broad associations with obesity and cardiovascular risk toward a more focused investigation of glucose metabolic dysregulation and preventive strategies.

**Figure 8:**
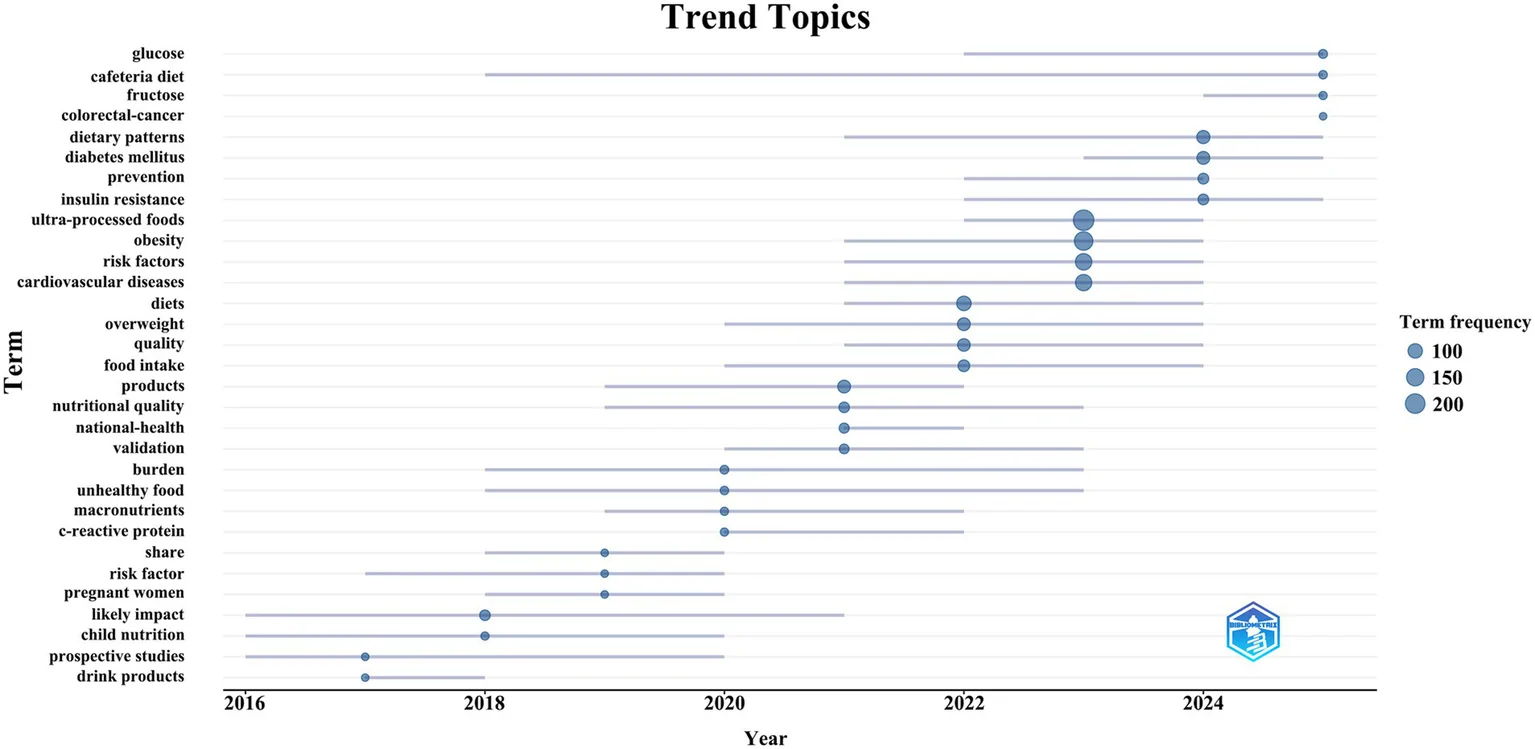
Trending topics in the field of ultra-processed food intake and cardiometabolic health.

### 3.5 Integrated analysis of research hotspots

An integrated examination of citation burst patterns, keyword occurrence frequencies, co-occurrence cluster structures, and longitudinal thematic shifts enabled the identification and consolidation of emerging research priorities in the domain of UPF intake and cardiometabolic health. The results collectively point to three predominant focal areas that characterize the current research landscape. (1) Association between UPF intake and CMDs: UPFs may increase the risk of CVD by promoting weight gain, inducing or exacerbating T2DM, dyslipidemia, and hypertension. (2) Association between UPF consumption and early cardiometabolic impairment in children and adolescents: UPFs may elevate the long-term risk of CMDs in children and adolescents by promoting obesity, shaping dietary habits, and inducing early disturbances in glucose and lipid metabolism. (3) Mechanisms underlying food matrix disruption and food additive-mediated cardiometabolic dysregulation: UPFs may promote cardiometabolic dysregulation through multiple pathways, including inflammatory responses, gut microbiota imbalance, impaired insulin signaling, and vascular endothelial dysfunction.

## 4 Discussion

### 4.1 General characteristics

To better characterize the research priorities and emerging trends in the field of UPF intake and cardiometabolic health, this study conducted a comprehensive bibliometric analysis with data visualization. A systematic search of the WoSCC identified 480 publications from 2015 to 2025. Analysis of publication trends revealed a steady increase in annual research output throughout this period. The most pronounced increase in WoSCC occurred between 2020 and 2021, whereas Scopus exhibited an additional increase between 2022 and 2023. This additional growth may reflect the rapid accumulation of evidence demonstrating associations between UPF intake and obesity, type 2 diabetes mellitus, cardiovascular diseases, and related metabolic risk factors, thereby underscoring the increasing scholarly interest and research activity in this field.

In terms of country distribution, Brazil led in publication output (*n* = 147), followed by the United States (*n* = 82) and China (*n* = 32). Among the top 20 most productive institutions, Brazil (7 institutions) and the United States (7 institutions) jointly dominated, followed by Iran (3 institutions), Spain (2 institutions), and Australia (1 institution), with the University of São Paulo ranking first with 89 publications. Brazil’s leading position in this field may be attributed to its theoretical primacy. Professor Carlos A. Monteiro and his team at the University of São Paulo developed the NOVA food classification system and formally defined the concept of UPFs, thereby establishing a globally recognized research paradigm and analytical framework for this field. Consequently, Brazil has accumulated a strong theoretical foundation, well-established national nutrition cohorts, and considerable academic influence in this research area. The United States represents another major contributor, supported by extensive national dietary surveys, large prospective cohorts, and strong institutional research capacity. These resources provide an important foundation for investigating the relationship between UPF intake and cardiometabolic outcomes. With exceptionally high UPF consumption and a substantial burden of obesity and cardiometabolic disease, the United States has emerged as a key setting for investigating the adverse health consequences of UPFs (27–29). China’s position among the leading contributors indicates growing research engagement in this field.

Analysis of the publication landscape revealed that the 480 included articles were distributed across 172 academic journals. Among these, *Nutrients*, *Public Health Nutrition*, and *Frontiers in Nutrition* served as the primary publication platforms in this field. Notably, *Nutrients* and *Public Health Nutrition* occupied the top two positions in both total citation counts and publication volume. This convergence not only reflects the broad recognition of the research published in these journals but also demonstrates their central role in establishing the field’s knowledge base and advancing its research frontiers. The predominance of nutrition- and public-health-oriented journals reflects the field’s origins in dietary classification, nutritional epidemiology, and population-level risk assessment. By contrast, the relatively limited participation of clinical cardiovascular journals may indicate that the translation of existing epidemiological evidence into cardiovascular clinical research remains insufficient. Future research should therefore place greater emphasis on translating the available evidence into effective disease-prevention strategies.

### 4.2 Research hotspots and emerging trends

As described above, through an integrated analysis of citation bursts, keyword frequency profiling, keyword co-occurrence clustering, and thematic evolution mapping, the emerging research hotspots in the field of UPF intake and cardiometabolic health were systematically identified. The findings strongly indicate that current frontier research in this field converges on three key directions: (1) association between UPF intake and CMDs; (2) association between UPF consumption and early cardiometabolic impairment in children and adolescents; and (3) mechanisms underlying food matrix disruption and food additive-mediated cardiometabolic dysregulation.

#### 4.2.1 Association between UPF intake and CMDs

Keyword co-occurrence clusters showed that “obesity,” “cardiovascular diseases,” “diabetes mellitus,” “hypertension,” and “metabolic syndrome” constituted the core themes in this field. References exhibiting strong citation bursts also focused on the associations between UPF intake and overweight, obesity, hypertension, and other cardiometabolic outcomes.

Existing evidence indicates a consistent association between UPF intake and the risk of CMDs. UPF consumption may increase the risk of CVD through multiple pathways, including promotion of weight gain and induction or exacerbation of T2DM, dyslipidemia, and hypertension.

Regarding effects on body weight, observational evidence has consistently associated higher UPF intake with weight gain and abdominal obesity (23, 30–33). In an RCT, the ultra-processed and unprocessed diets offered were matched for energy content and macronutrient composition; however, under ad libitum conditions, participants consumed approximately 500 kcal/day more during the ultra-processed diet phase, accompanied by gains of approximately 0.9 kg in body weight and 0.4 kg in body fat over 2 weeks (34).

Regarding effects on glucose metabolism, multiple cohort studies and meta-analyses have indicated that total UPF intake is associated with a higher risk of T2DM (35–37). In a U.S. population-based cohort, comparison of the highest versus lowest quintiles of total UPF intake yielded a hazard ratio of 1.46 for T2DM (95% CI: 1.39–1.54) (36). The same study identified positive associations between type 2 diabetes risk and the consumption of ultra-processed refined grains, animal-based products, artificially sweetened and sugar-sweetened beverages, ready-to-eat or ready-to-heat mixed dishes, and sauces, spreads, and condiments (36).

Regarding effects on lipid metabolism, high UPF intake has been significantly associated with incident hypertriglyceridemia (≥150 mg/dL). Among individuals free of dyslipidemia at baseline, those in the highest tertile of energy intake from UPFs had 2.66 times the odds of developing hypertriglyceridemia compared with those in the lowest tertile (38).

Regarding effects on blood pressure, high UPF consumption was found to significantly increase the risk of incident hypertension (37, 39). Cohort data further demonstrated a dose–response relationship, with hypertension incidence rising progressively across quartiles of UPF intake (40). Additionally, higher body mass index and diet quality were found to mediate the association between UPF intake and hypertension incidence differently in Black and White adults, a relationship that may partly be attributed to excessive dietary sodium intake, which elevates blood pressure through impaired renal sodium handling, sodium and water retention, and expansion of blood volume (40, 41).

Beyond individual metabolic risk factors, UPF intake has also been closely associated with hard cardiovascular endpoints, including cardiovascular events and mortality. Evidence from cohort studies and meta-analyses indicates that increasing UPF consumption is linearly associated with cardiovascular event risk, while its association with all-cause mortality follows a non-linear pattern (42–45).

Overall, higher UPF intake has been associated with multiple cardiometabolic risk factors and clinical outcomes. Potential pathways include excess energy intake, adiposity, insulin resistance, dyslipidemia, and altered blood pressure regulation, although the causal relationships require further investigation.

#### 4.2.2 Association between UPF consumption and early cardiometabolic impairment in children and adolescents

The keyword clusters were characterized by terms such as “children,” “adolescents,” “obesity,” “overweight,” and “food intake,” while references exhibiting citation bursts also addressed lipid metabolism, overweight, and obesity in children. These findings indicate that early-life exposure to UPFs and their potential long-term cardiometabolic consequences have become important research hotspots in this field.

UPFs have become increasingly prevalent in modern diets, driven by their convenience, palatability, attractive appearance, and effective marketing strategies, all of which contribute to their widespread acceptance and high consumption among children and adolescents (46–49). Analysis of nationally representative data demonstrated that the majority of daily caloric intake among U.S. adolescents is derived from UPFs, with the estimated proportion of energy provided by UPFs rising from 61.4% in 1999 to 67.0% in 2018 (50). Evidence suggests that UPFs may begin to exert dysmetabolic effects as early as childhood (51). Early UPF intake has been closely associated with increased obesity risk in children; studies have shown that UPF consumption in preschool-aged children predicts subsequent waist circumference gain during school age, and that higher UPF intake is significantly and positively associated with body mass index, fat mass index, and central adiposity in children (51, 52).

Dietary preferences and habits established in early childhood are of critical importance, as they tend to persist and may exert lasting influence through adolescence and into adulthood (53, 54). On one hand, the characteristically high energy density, soft texture, and rapid consumption rate of UPFs may attenuate satiety signaling (55, 56). On the other hand, the intense reward stimulation generated by flavor-enhancing additives in UPFs may dysregulate feeding-related neural circuits, thereby facilitating overconsumption (57). Furthermore, individuals with UPF-addictive eating patterns have been reported to experience withdrawal-like symptoms upon attempting to reduce UPF intake, including headaches, fatigue, irritability, and intense cravings (58). Abdominal obesity associated with UPF consumption at age 8 years has been identified as a potential risk factor for subsequent impaired glucose metabolism, thereby increasing the probability of CVD in adulthood (51). UPF intake has additionally been shown to adversely affect lipid metabolism in children, with potential implications for future CVD risk. A longitudinal study found that children with higher UPF consumption at age 3 years had significantly elevated levels of total cholesterol and low-density lipoprotein cholesterol by age 6 years (59).

Overall, childhood and adolescence may represent important periods of susceptibility to UPF-related cardiometabolic risk. However, the available evidence is predominantly observational, and the long-term causal effects remain uncertain.

#### 4.2.3 Mechanisms underlying food matrix disruption and food additive-mediated cardiometabolic dysregulation

Terms identified through the keyword clustering and trend-topic analyses, including “insulin resistance,” “inflammation,” “gut microbiota,” “oxidative stress,” and “artificial sweeteners,” indicate that research is increasingly focusing on the biological pathways through which food matrix alterations and food additives may influence cardiometabolic health.

Industrial processing may partly contribute to the adverse health effects associated with UPFs by disrupting the native food matrix and introducing food additives (60). These characteristics may influence energy intake, satiety, inflammation, the gut microbiota, insulin signaling, and endothelial function.

In unprocessed whole plant foods, nutrients are typically encased within intact plant cell walls, protein networks, and dietary fiber structures (61, 62). This native food matrix slows nutrient release, increases the oral processing load, and enhances satiety (61, 63). In contrast, the extensive industrial processing of UPFs, including ultra-fine milling, extrusion, reconstitution, and refining, severely disrupts this matrix, rendering nutrients far more susceptible to rapid digestion and absorption (64). Matrix disruption reduces oral processing time during eating, may accelerate gastric emptying, and increases the efficiency of nutrient absorption, thereby facilitating excess energy intake (65–67). Studies indicate that ultra-processed diets may increase ad libitum energy intake and eating rate, potentially due to altered food structure and reduced oro-sensory exposure associated with industrial processing (34, 68). These accelerated eating and gastric emptying rates impair nutrient sensing in the distal gastrointestinal tract, thereby disrupting the secretion of key incretin and satiety hormones, including glucagon-like peptide-1 and peptide YY, and delaying the generation of satiety signals (69, 70). Rapid nutrient absorption also induces pronounced postprandial fluctuations in blood glucose and insulin levels, compounding metabolic load and promoting insulin resistance. Overall, current evidence consistently supports that ultra-processed diets increase energy intake; however, the independent contribution of food matrix disruption to the long-term onset and progression of CMDs remains insufficiently supported by high-quality longitudinal studies.

Beyond food matrix disruption, food additives widely used in UPFs may also contribute to metabolic dysregulation. Current research has focused primarily on emulsifiers, artificial sweeteners, and certain preservatives.

Emulsifiers are widely used to improve food texture and stability. Emerging evidence suggests that certain emulsifiers may contribute to cardiometabolic abnormalities by disrupting intestinal permeability, gut microbiota composition, and insulin signaling pathways. Carboxymethylcellulose, polysorbate-80, and carrageenan have been shown to alter gut microbial composition, compromise intestinal mucus layer integrity, and increase intestinal permeability, thereby inducing metabolic endotoxemia and chronic low-grade inflammation (71–75). In overweight individuals, carrageenan exposure was associated with reduced systemic and hepatic insulin sensitivity, accompanied by elevated inflammatory markers, including C-reactive protein and interleukin-6, and increased intestinal permeability (73). Removal of dietary carrageenan improved glycated hemoglobin and homeostatic model assessment of insulin resistance levels in prediabetic participants, implicating this additive in insulin signaling impairment and glucose intolerance (76). Mechanistic studies further indicate that carrageenan may interfere with insulin receptor binding and suppress phosphoinositide 3-kinase/protein kinase B pathway (PI3K/Akt) activation, thereby impairing insulin signal transduction (77). Prospective cohort data have additionally linked long-term consumption of several common emulsifiers to an elevated risk of T2DM (78, 79). Collectively, available evidence suggests that emulsifiers may promote obesity, insulin resistance, and T2DM, although the precise underlying mechanisms warrant further investigation.

Artificial sweeteners are non-nutritive additives commonly used in UPFs to provide sweetness without increasing caloric intake. Compounds such as aspartame, sucralose, saccharin, and acesulfame-K may contribute to cardiometabolic injury through multiple mechanisms, including modulation of gut microbiota, glucose metabolism, insulin signaling, and vascular inflammation. Artificial sweeteners have been shown to shift gut microbial composition toward an obesity-associated phenotype and to induce chronic low-grade inflammation, thereby further promoting insulin resistance and obesity (80). Aspartame may additionally activate intestinal sweet taste receptors and vagal pathways to induce aberrant insulin secretion, establishing a state of sustained hyperinsulinemia that exacerbates systemic insulin resistance (81). Furthermore, aspartame may act directly on vascular endothelial cells, upregulating membrane-bound chemokine CX3CL1 expression and promoting the recruitment and M1 pro-inflammatory polarization of CX3CR1-positive monocytes and macrophages at the vessel wall, thereby amplifying local inflammatory responses and accelerating atherosclerosis progression (81). Multiple prospective studies have found that artificial sweetener intake is associated with increased risks of overall CVD, heart failure, and T2DM, with particularly pronounced associations observed for cerebrovascular disease risk (82, 83). Further analysis revealed that aspartame intake was specifically associated with an elevated risk of cerebrovascular events, while acesulfame potassium and sucralose were primarily linked to increased coronary heart disease risk (84). T2DM may partially mediate the association between artificial sweetener consumption and CVD, suggesting that glucose metabolic dysregulation represents an important intermediate mechanism in artificial sweetener-promoted cardiovascular injury (82). Overall, the long-term cardiometabolic effects of artificial sweeteners require further clarification.

Preservatives, in addition to extending food shelf life, have increasingly been implicated in cardiometabolic dysregulation. Among the most studied are nitrites, nitrates, and parabens. Notably, the biological effects of nitrates and nitrites differ markedly by source. Dietary nitrates naturally present in dark green leafy vegetables enhance nitric oxide bioavailability via the nitrate–nitrite–nitric oxide pathway, improving endothelial function, promoting vasodilation, and attenuating oxidative stress, with some evidence suggesting protective effects against obesity, T2DM, hypertension, and metabolic syndrome (85–90). In contrast, nitrite exposure from food additives, particularly sodium nitrite in processed meats, may exert opposing effects. A prospective cohort study associated higher intake of additive-derived nitrites with an approximately 19% higher risk of hypertension, whereas nitrates and nitrites from natural food and water sources showed no comparable association. These findings suggest that the observed relationship may vary according to the source of exposure (91). Parabens represent another class of preservatives that have attracted considerable research attention. These compounds possess weak estrogenic activity and are classified as endocrine-disrupting chemicals, potentially contributing to metabolic abnormalities through effects on lipid metabolism, inflammatory responses, and insulin signal transduction. Elevated levels of methylparaben and propylparaben have been observed in overweight and obese women. The underlying mechanism may involve paraben-mediated activation of peroxisome proliferator-activated receptor gamma, promoting adipocyte differentiation and lipid accumulation while impairing glucose uptake and insulin sensitivity (92). Additional studies have reported associations between methylparaben, propylparaben, and butylparaben exposure and insulin levels and insulin resistance, although heterogeneity and non-linear effects across compounds have been noted (93). Parabens may also induce chronic low-grade inflammation through modulation of inflammatory mediators including interleukin-1β, interleukin-6, tumor necrosis factor alpha, and transforming growth factor beta (93). Furthermore, elevated urinary levels of ethylparaben, propylparaben, and total parabens have been associated with increased hypertension risk, with *ESR1* gene polymorphisms potentially modifying this association (94); however, the overall evidence base remains limited, and the significance of parabens as a meaningful contributor to UPF-related cardiometabolic risk requires further investigation. Available evidence suggests possible associations between certain preservatives and obesity, insulin resistance, and hypertension, but remains limited and heterogeneous.

### 4.3 Limitations

This study systematically mapped the research progress, hotspots, and trends in the field of UPF intake and cardiometabolic health using the Web of Science and Scopus databases, providing an important reference for deepening understanding of this field and guiding future research directions. Nevertheless, several limitations should be acknowledged. First, the data sources were restricted to these two databases; although Web of Science is widely recognized as an ideal tool for bibliometric analysis owing to its rigorous quality standards, the exclusion of other databases may have resulted in the omission of some relevant publications. Second, the inclusion of only English-language articles and review articles may have introduced language bias and document-type selection bias. Studies from non-English-speaking countries or regions, as well as conference papers, dissertations, book chapters, and institutional reports, may therefore have been excluded. Third, WoSCC and Scopus differ in journal coverage, data fields, cited-reference formats, and citation-counting rules. To avoid metadata inconsistencies or the loss of critical information during database merging, the two databases were analyzed separately; however, this approach limited the direct integration of their results. Fourth, although the literature search was completed on March 19, 2026, some publications from 2025 may not yet have been fully included or indexed in WoSCC or Scopus. Consequently, publication output for 2025 may have been underestimated, and keyword frequencies and trend topics for the most recent year may not yet be stable. Fifth, the analytical approaches employed in CiteSpace and VOSviewer cannot fully substitute for systematic literature retrieval, and bibliometric methods are subject to inherent limitations, including the inability to assess the quality of individual studies and the time-dependent nature of citation metrics, whereby recently published articles may be underrepresented in citation counts due to insufficient accumulation time. Sixth, the results of keyword clustering and trend identification may be influenced by keyword standardization rules, minimum occurrence thresholds, and software parameter settings; therefore, the identified research hotspots and thematic boundaries are method-dependent to some extent. Overall, the findings of this study are primarily intended to describe the general research structure, major hotspots, and potential future directions in the field of UPF intake and cardiometabolic health. Further high-quality studies conducted by a broader range of research teams are needed to elucidate the mechanisms underlying the association between UPFs and cardiometabolic health, establish causal relationships, and develop targeted intervention strategies.

## 5 Conclusion

Using records retrieved from the WoSCC and Scopus databases, this study systematically mapped the scholarly literature concerning UPF intake and cardiometabolic health over the decade from 2015 to 2025 through bibliometric methods. The findings reveal a sustained and rapid growth in research output over the past decade, with Brazil and the United States dominating global publication volume and institutional contribution. The University of São Paulo, in particular, has played a central and leading role in the global academic network. The convergence of high publication output and high citation counts in core journals such as *Nutrients* and *Public Health Nutrition* underscores their dual role in both establishing the knowledge base and driving the frontier development of this field.

By synthesizing evidence from burst detection, co-occurrence network analysis, and longitudinal thematic mapping, three dominant areas of research focus were delineated. Available epidemiological and interventional evidence indicates that higher UPF intake is associated with heightened susceptibility to obesity, T2DM, dyslipidemia, hypertension, cardiovascular events, and all-cause mortality, with detrimental effects observed across multiple biological targets and pathways. Second, children and adolescents represent a high-risk population for UPF exposure; early consumption may exert lasting adverse effects on adult cardiometabolic health by promoting obesity, inducing early disturbances in glucose and lipid metabolism, and reinforcing unhealthy dietary habits. Third, food additives, including emulsifiers, artificial sweeteners, and nitrite-containing preservatives, may contribute to cardiometabolic dysregulation through multiple mechanisms, including gut microbiota imbalance, impaired insulin signaling, chronic inflammation, and endothelial dysfunction, while food matrix disruption-induced rapid nutrient absorption and attenuated satiety signaling also represent important pathogenic mechanisms.

The knowledge map constructed in this study provides a reference framework for researchers, clinicians, and public health practitioners in this field. Future research should focus on establishing causal evidence through high-quality prospective studies and intervention trials, systematically comparing the differential effects of specific UPF subgroups and individual food additives, and developing mechanism-based targeted dietary intervention strategies, with the overarching goal of providing a scientific foundation for reducing the global burden of CMDs.

## Data Availability

Publicly available datasets were analyzed in this study. This data can be found at: Web of Science: https://www.webofscience.com/wos/; Scopus: https://www.elsevier.com/products/scopus.

## Supplementary material

SUPPLEMENTARY APPENDIX 1 Full search strategies for the Web of Science Core Collection and Scopus databases.

SUPPLEMENTARY FIGURE S1 Annual publication output of research on ultra-processed food intake and cardiometabolic health in the Scopus database, 2015–2025.

SUPPLEMENTARY FIGURE S2 Distribution of the top 20 corresponding-author countries in the Scopus dataset.

SUPPLEMENTARY FIGURE S3 Keyword co-occurrence network of publications based on the Scopus dataset.

SUPPLEMENTARY TABLE S1 Complete list of keywords and corresponding cluster assignments derived from the Scopus keyword co-occurrence analysis.
