# Supplementary material for: Global research landscape and emerging frontiers of ultra-processed food intake and cardiometabolic health: a bibliometric and knowledge-mapping analysis (2015–2025)
Source: Front Nutr. 2026 Oct 7;13:1916253. doi: 10.3389/fnut.2026.1916253 (PMC13684074; doi:10.3389/fnut.2026.1916253)
Supplement: Supplementary file 1 [file Table_1.DOCX]

**The search strategy for the Web of Science Core Collection was as follows:**

TS=("ultra-processed food*" OR "ultraprocessed food*" OR "ultra-processed diet*" OR UPF OR UPFs OR "NOVA classification") AND TS=("cardiovascular disease*" OR "hypertension" OR "blood pressure" OR "endothelial dysfunction" OR "atherosclerosis" OR "cardiometabolic" OR "obesity" OR "diabetes" OR "Metabolic Syndrome" OR "Syndrome, Metabolic" OR "Insulin Resistance Syndrome" OR "Dysmetabolic Syndrome" OR "Reaven Syndrome" OR "Metabolic Cardiovascular Syndrome" OR "dyslipidemia*" OR "hyperlipidemia*" OR "hypercholesterolemia*" OR "hypertriglyceridemia*" )

**The search strategy for the Scopus was as follows:**

TITLE-ABS-KEY ( "ultra-processed food*" OR "ultraprocessed food*" OR "ultra-processed diet*" OR UPF OR UPFs OR "NOVA classification" ) AND TITLE-ABS-KEY ( "cardiovascular disease*" OR "hypertension" OR "blood pressure" OR "endothelial dysfunction" OR "atherosclerosis" OR "cardiometabolic" OR "obesity" OR "diabetes" OR "Metabolic Syndrome" OR "Syndrome, Metabolic" OR "Insulin Resistance Syndrome" OR "Dysmetabolic Syndrome" OR "Reaven Syndrome" OR "Metabolic Cardiovascular Syndrome" OR "dyslipidemia*" OR "hyperlipidemia*" OR "hypercholesterolemia*" OR "hypertriglyceridemia*" ) AND PUBYEAR > 2014 AND PUBYEAR < 2026 AND ( LIMIT-TO ( DOCTYPE , "ar" ) OR LIMIT-TO ( DOCTYPE , "re" ) ) AND ( LIMIT-TO ( LANGUAGE , "English" ) )
